# The Complexity of Two Colouring Games

**DOI:** 10.1007/s00453-022-01069-w

**Published:** 2022-11-24

**Authors:** Stephan Dominique Andres, François Dross, Melissa A. Huggan, Fionn Mc Inerney, Richard J. Nowakowski

**Affiliations:** 1 Institute of Mathematics and Computer Science, University of Greifswald, Greifswald, Germany; 2 CNRS, Bordeaux INP, LaBRI, UMR5800, Univ. Bordeaux, 33400 Talence, France; 3 Department of Mathematics, Vancouver Island University, Nanaimo, BC Canada; 4 CISPA Helmholtz Center for Information Security, Saarbrücken, Germany; 5 Department of Mathematics and Statistics, Dalhousie University, Halifax, NS Canada

**Keywords:** Orthogonal colouring game, Orthogonal graph colouring, Combinatorial game, Scoring game, Strictly matched involution, PSPACE-complete, NP-complete

## Abstract

We consider two variants of *orthogonal colouring games* on graphs. In these games, two players alternate colouring uncoloured vertices (from a choice of $m\in {\mathbb {N}}$ colours) of a pair of isomorphic graphs while respecting the properness and the orthogonality of the partial colourings. In the *normal play variant*, the first player unable to move loses. In the *scoring variant*, each player aims to maximise their *score*, which is the number of coloured vertices in their copy of the graph. We prove that, given an instance with partial colourings, both the normal play and the scoring variant of the game are PSPACE-complete. An involution $\sigma$ of a graph *G* is *strictly matched* if its fixed point set induces a clique and $v\sigma (v)\in E(G)$ for any non-fixed point $v\in V(G)$. Andres et al. (Theor Comput Sci 795:312–325, 2019) gave a solution of the normal play variant played on graphs that admit a strictly matched involution. We prove that recognising graphs that admit a strictly matched involution is NP-complete.

## Introduction

Graph colouring games have received significant attention since the early 1990’s when Bodlaender reintroduced the *vertex colouring game* of Brams [15] under the name of the *colouring construction game* [9]. In this game, there is a graph *G* whose vertices are initially uncoloured, and two players, Alice and Bob, take turns colouring an uncoloured vertex of *G* from a set of $k\ge 1$ colours, while maintaining that the partial colouring is *proper*, i.e., any two adjacent coloured vertices have distinct colours. Alice wins if all of the vertices of *G* are eventually coloured, while Bob wins otherwise. The *game chromatic number* of a graph *G*, denoted by $\chi _g(G)$, is the minimum number of colours $k\ge 1$ such that Alice has a winning strategy in the colouring construction game. The *game chromatic number* $\chi _g({{\mathcal {G}}})$ of a non-empty class ${{\mathcal {G}}}$ of graphs is defined as

$$\begin{aligned}\chi _g({{\mathcal {G}}})=\sup \limits _{G\in {{\mathcal {G}}}}\chi _g(G).\end{aligned}$$

Much is known about the game chromatic number for different graph classes such as the class ${\mathcal {F}}$ of forests ($\chi _g({\mathcal {F}})=4$) [14], the class ${\mathcal {C}}$ of cacti ($\chi _g({\mathcal {C}})=5$) [26], the class ${\mathcal {O}}$ of outerplanar graphs ($6\le \chi _g({\mathcal {O}})\le 7$) [16], and the class ${\mathcal {P}}$ of planar graphs ($7\le \chi _g({\mathcal {P}})\le 17$) [5, 28], to name a few. The game has attracted significant attention due to its ties to graph colouring, which is one of the classic problems in graph theory, but also due in part to two very interesting long-standing open questions.

The first question was posed by Bodlaender in 1991 in [9]. Given a graph *G* and an integer $k\ge 3$, it asks to determine the computational complexity of deciding whether $\chi _g(G)\le k$. It took almost 30 years for any progress to be made on this question, with Costa et al. only recently partially answering it in [11], where they showed that deciding if $\chi _g(G)\le k$ is PSPACE-complete when *k* is part of the input. However, the question remains open when *k* is a fixed constant.

The second question, posed by Zhu in 1999 in [27], asks whether, for a given graph *G*, Alice has a winning strategy with $k+1$ colours in *G* if she has a winning strategy with *k* colours in *G*. This question is particularly intriguing since, intuitively, one would think that having more colours is advantageous to Alice, but this question still remains open to this day.

There are also many other colouring games on graphs that have been studied. Some of them, e.g., the *edge colouring game* [17] and the *incidence colouring game* [1], can be considered as special cases of the vertex colouring game. Others, such as the *colouring game* [9, 24], the *marking game* [27], and the *orthogonal colouring game* [2], rely on different colouring concepts. This paper focuses on the latter game and a variant thereof.

The concept of orthogonality in graph theory is motivated by the orthogonality of Latin squares, which is a major topic in finite geometry. Orthogonal edge colourings of graphs were already considered by Archdeacon et al. [3] in 1985. In this paper, we consider orthogonal vertex colourings, introduced by Caro and Yuster [10] in 1999, which strictly generalise the concept of orthogonality of Latin squares (cf. Ballif [4]). Recall that a partial colouring of a graph $G=(V,E)$ is *proper* if any two adjacent coloured vertices have distinct colours. Two partial colourings $c_A$ and $c_B$ of *G* are *orthogonal* if, for any two vertices $v,w\in V$ that are coloured in both $c_A$ and $c_B$, the ordered pair of colours of *v* differs from the ordered pair of the colours of *w*, i.e.,

$$\begin{aligned} (c_A(v),c_B(v))\ne (c_A(w),c_B(w)). \end{aligned}$$

In this paper, we consider two game-theoretic variants of orthogonal graph colouring. Both games are played on two isomorphic copies $G_A$ and $G_B$ of a given graph *G* by two players, Alice and Bob. We identify the vertices of $G_A$ and $G_B$ with their preimages in *G*. Initially, every vertex is uncoloured. Alternately, the players choose either $G_A$ or $G_B$, and colour one of its uncoloured vertices with a colour from the set $\{1,\ldots ,m\}$, thus creating partial colourings $c_A$ and $c_B$ of *G*, such that the properness and orthogonality of the partial colourings are not violated. The game ends when the players are unable to move. We call the general framework of this type of game the *orthogonal colouring game*. The winning conventions of the two variants of the game differ.

In the *normal play variant* $NorMOC_m(G)$ of the orthogonal colouring game played with *m* colours, the first player unable to move loses, and the other player wins. So, there is no possibility of a draw.

In the *scoring variant* $MOC_m(G)$ of the orthogonal colouring game played with *m* colours, Alice owns $G_A$ and Bob owns $G_B$. A player’s *score* is the number of coloured vertices in their copy of *G*. When the players are unable to move, the player with the higher score wins. If the scores are equal, there is a draw.

These games were introduced by Andres et al. [2] who called the scoring variant simply *orthogonal colouring game*. The scoring variant is particularly interesting since it is, to the best of our knowledge, the first game that is both a graph colouring game and a scoring game. As we have seen, graph colouring games have been vastly studied, but scoring games are also very well-studied in combinatorial game theory. Scoring game theory was introduced in the 1950’s by Milnor [21], but has only recently (in the last 10–20 years) gained a lot of attention. The papers [18, 19] introduced some theory around scoring games in general, and there have been many papers as of late focusing on different scoring games (see, e.g., [7, 8, 13, 20, 25]).

In the scoring variant of the orthogonal colouring game, it is worth noting that playing in the adversary’s copy of *G* may be advantageous in some cases. It may even lead to a win, as the example of the game on the 4-cycle $C_4$ played with two colours shows, which is won by Bob [2]. Indeed, Bob’s strategy to win with a score that is 2 greater than Alice’s score is as follows: when Alice plays on her first move on a $C_4$, Bob responds in the same $C_4$ on the non-adjacent vertex, colouring with the opposite colour if it is her $C_4$ and the same colour if it his $C_4$ [2].

The main results of [2] are the introduction and characterisation of a class of graphs, called *graphs admitting a strictly matched involution*, where it is proven that Bob has a strategy to guarantee a draw in the scoring variant of the orthogonal colouring game.

In this paper, we answer several open questions regarding the complexity of the two variants of the orthogonal colouring game. We first consider the complexity of the two variants of the game when partial colourings are given as part of the input before the game starts. Moreover, we denote the normal play variant on a graph *G* with *m* colours and given initial partial colourings $c_A$ and $c_B$ of $G_A$ and $G_B$, respectively, by $NorMOC_m(G,c_A,c_B)$, and the scoring variant on a graph *G* with *m* colours and given initial partial colourings $c_A$ and $c_B$ of $G_A$ and $G_B$, respectively, is denoted by $MOC_m(G,c_A,c_B)$. Thus, an instance $(G,c_A,c_B)$ of the games consists of a graph *G* and initial partial colourings $c_A$ and $c_B$ of $G_A$ and $G_B$, respectively.

We prove the PSPACE-completeness of the normal play variant $NorMOC_m(G,c_A,c_B)$ using the PSPACE-completeness of the Proper *m* colouring game proved by Schaefer [24] for $m=1$ (in which case the game is known as Node Kayles) and by Beaulieu et al. [6] for every $m\ge 2$:

### Theorem 1

Given an instance $(G,c_A,c_B)$, the problem of determining the outcome of the normal play variant $NorMOC_m(G,c_A,c_B)$ of the orthogonal colouring game under optimal play is PSPACE-complete for all $m\ge 1$.

A reduction from QSAT (shown to be PSPACE-complete by Schaefer [23]) is given to prove the scoring variant $MOC_m(G,c_A,c_B)$ is PSPACE-complete:

### Theorem 2

Given an instance $(G,c_A,c_B)$, the problem of determining the outcome of the scoring variant $MOC_m(G,c_A,c_B)$ of the orthogonal colouring game under optimal play is PSPACE-complete for all $m\ge 3$.

Such results are quite interesting since, as we have seen with the colouring construction game, determining the complexity of colouring games in general is very difficult. Moreover, the complexity of many scoring games is not known, as they can be notoriously difficult to analyse, so our results are novel with respect to this aspect as well. We conclude by proving that the recognition of graphs admitting a strictly matched involution is an NP-complete problem.

The paper is structured as follows. In Sect. 2, we introduce notation and define graphs that admit a strictly matched involution. The proof of the PSPACE-completeness of the normal play variant of the orthogonal colouring game with partial colourings as part of the input is given in Sect. 3, which is then followed by the proof of the PSPACE-completeness of the scoring variant with partial colourings as part of the input in Sect. 4. The NP-completeness of the recognition of graphs admitting a strictly matched involution is proven in Sect. 5. In Sect. 6, we conclude with some open problems.

## Notation and Definitions

First, we fix some general notation. We use standard notation from computational complexity theory (see, e.g., [22]). All graphs we consider are simple and undirected, and we use standard notation from graph theory (see, e.g., [12]). By $K_n$ ($C_n$, respectively) we denote the complete graph (cycle, respectively) on *n* vertices. The graph $2K_2$ consists of two disjoint copies of a $K_2$, and the graph $K_1\cup K_2$ is the graph on three vertices with exactly one edge.

Let $G=(V,E)$ be a graph. An *involution* of *G* is a graph automorphism $\sigma :V\longrightarrow V$ of *G* that satisfies

$$\begin{aligned}\sigma \circ \sigma =\textrm{id}_{V},\end{aligned}$$

where $\textrm{id}_V$ is the identity on *V*. An involution $\sigma$ of *G* partitions the vertex set *V* into two sets: the *fixed point set*

$$\begin{aligned}F_1(G)=\{v\in V\mid \sigma (v)=v\}\end{aligned}$$

and the set

$$\begin{aligned}F_2(G)=V\setminus F_1(G)\end{aligned}$$

of vertices in 2-orbits of *V* under the action of $\sigma$. Andres et al. [2] defined an involution $\sigma$ of *G* to be *strictly matched* if

(SI 1): the set $F_1(G)$ induces a (possibly empty) complete graph and,
(SI 2): for every $v\in F_2(G)$, we have the (matching) edge $v\sigma (v)\in E$.

In an orthogonal colouring game played with *m* colours on copies $G_A$ and $G_B$ of a graph $G=(V,E)$, an *orthogonal pair* is an ordered pair $(s_A,s_B)$ of colours $s_A,s_B\in \{1,\ldots ,m\}$, such that there exists a vertex $v\in V$ with $c_A(v)=s_A$ and $c_B(v)=s_B$, where $c_A$ ($c_B$, respectively) corresponds to the partial colouring of the isomorphic copy $G_A$ ($G_B$, respectively) of *G*.

## Complexity of the Normal Play Orthogonal Colouring Game

In this section, we show that the normal play variant of the orthogonal colouring game is PSPACE-complete when partial colourings of the graph are given as part of the input. The reduction is from the Proper *m* colouring game under normal play convention, which is the following:

### Definition 3

(Proper *m* colouring game) Given an initially uncoloured graph *G*, two players, Alice and Bob, take turns colouring the uncoloured vertices of *G* with one of *m* colours while maintaining that the colouring is proper. Alice goes first and only one vertex may be coloured by a player on their turn. The first player who cannot colour a vertex loses.

For this game, the problem is to determine whether Alice has a winning strategy. As already mentioned, the following is well-known.

### Theorem 4

(Schaefer [24]) The Proper *m* colouring game is PSPACE-complete for $m=1$.

### Theorem 5

(Beaulieu et al. [6]) The Proper *m* colouring game is PSPACE-complete for any $m\ge 2$.

The hardness proof of Theorem 1 consists of reducing the Proper *m* colouring game to the game $NorMOC_m(G,c_A,c_B)$, the normal play variant of the orthogonal colouring game with given initial partial colourings.

### Proof of Theorem 1

For fixed *m*, by a game-tree search, the problem $NorMOC(G,c_A,c_B)$ can be solved for any instance $(G,c_A,c_B)$. We need only memorise the nodes on the path from the root to the actual node. Since the number of turns and possible plays is bounded above by the number of uncoloured vertices remaining, the problem is in PSPACE.

**Fig. 1:**
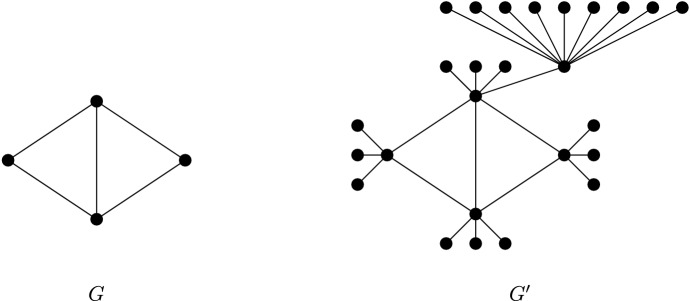
Example of the construction of $G'$ from a graph *G* for $m=3$

Now we proceed to prove the PSPACE-hardness of the problem by a reduction from the Proper *m* colouring game. Then the result follows by Theorem 4 for $m=1$ or Theorem 5 for $m\ge 2$. Let *G* be an instance of the Proper *m* colouring game. We construct an instance $(G',c'_A,c'_B)$ for the problem $NorMOC_m(G',c'_A,c'_B)$. From *G* we construct a new graph $G'$. The informal construction of $G'$ is as follows. For one vertex $v_n$ of *G*, add $m+1$ new adjacent vertices and, for the rest of the vertices of *G*, add *m* new adjacent vertices. For one of these new vertices adjacent to $v_n$, $v_n^{m+1}$, add $m^2$ new adjacent vertices. See Fig. 1 for an example.

**Fig. 2:**
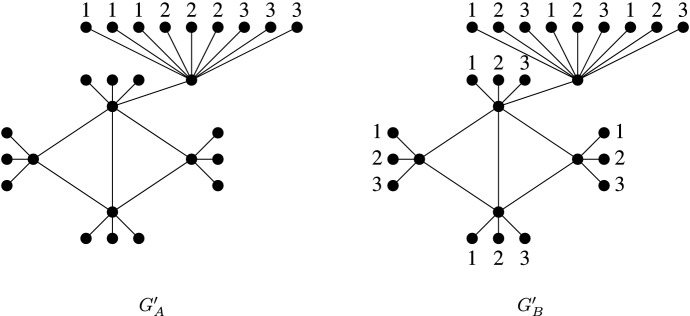
The initial partial colourings of $G_A'$ and $G_B'$

In the copies $G_A'$ and $G_B'$ of $G'$, the $m^2$ new vertices adjacent to $v_n^{m+1}$ are all coloured such that all possible ordered pairs of two colours exist between two corresponding vertices in two different copies of $G'$, and thus, all possible ordered pairs (*a*, *b*) of two colours of a vertex with colour *a* in $G_A'$ and colour *b* in $G_B'$ are used (i.e., they are orthogonal pairs). For each vertex of *G* in $G_B'$, all of the *m* new adjacent vertices are coloured with the colours 1 through *m*, and, in the case of $v_n$, $v_n^{m+1}$ is left uncoloured. See Fig. 2 for an example.

Now, none of the new vertices added may be coloured either because they are already coloured or due to orthogonality. None of the vertices in $G_B'$ may be coloured since they are already coloured or they are adjacent to every other possible colour. The only vertices that may be coloured are the vertices of *G* in $G_A'$. Note that none of these vertices have been played on in $G_B'$, and so, orthogonality may not prevent any of these vertices from being coloured any of the *m* colours. Furthermore, none of the neighbours of the vertices of *G* in $G_A'$ have been coloured, and thus, every move in the orthogonal colouring game is now equivalent to a move in the Proper *m* colouring game.

Formally, given an instance of the Proper *m* colouring game, $G=(V,E)$, create a new graph $G'=(V',E')$ as follows. Let $V=\{v_1,\ldots ,v_n\}$. Then,

$$\begin{aligned} V'= & {} V\cup \bigcup \limits _{i=1}^n\{v_i^1,\ldots ,v_i^m\}\cup \{v_n^{m+1}\}\cup \{v_n^{m+1,1},\ldots ,v_n^{m+1,m^2}\}.\\ E'= & {} E\cup \bigcup \limits _{i=1}^n \{(v_i,v_i^j):j\in \{1,\ldots , m\}\}\cup \{(v_n,v_n^{m+1})\}\\{} & {} \quad \cup \{(v_n^{m+1},v_n^{m+1,\ell }):\ell \in \{1,\ldots , m^2\}\}. \end{aligned}$$

For all $j,q\in {\mathbb {N}}$ with $1\le j\le m$ and $0\le q\le m-1$, the vertices $\{v_n^{m+1,qm+1},\ldots ,v_n^{m+1,qm+m}\}$ are coloured $q+1$ in $G_A'$ and the vertex $v_n^{m+1,qm+j}$ is coloured *j* in $G_B'$ (the other copy of $G'$). This results in every permutation of a pair of colours being forbidden by orthogonality. Now, for all *i*, *j* ($i\in {\mathbb {N}}$, $1\le i\le n$), the vertex $v_i^j$ is coloured *j* in $G_B'$. Clearly, the construction of $G'$ is achieved in polynomial time.

Note that both $G_A'$ and $G_B'$ have partial colourings that are proper and their partial colourings are orthogonal. Also, it is no longer possible to colour any vertices of $G_B'$ since the remainder of the uncoloured vertices are adjacent to at least one vertex of each of the *m* possible colours. Finally, it is not possible to colour any more of the new vertices added in $G_A'$ due to orthogonality, and only the new vertices non-adjacent to the vertices of *G* in $G_A'$ have been coloured. Therefore, only the uncoloured vertices of *G* in $G_A'$ may be coloured, and orthogonality cannot prevent any of the *m* colours from being used on these vertices as none of these vertices have been coloured in $G_B'$. Thus, every move in this instance of the orthogonal colouring game is now equivalent to a move in the given instance of the Proper *m* colouring game. $\square$

## Complexity of the Scoring Orthogonal Colouring Game

In this section, we show that the scoring variant of the orthogonal colouring game is PSPACE-complete when partial colourings of the graph are given as part of the input. The reduction is from the QSAT problem which is the following.

### Definition 6

(*QSAT*) Given a set of boolean variables $x_1,\ldots ,x_n$, a boolean formula

$$\begin{aligned}F=C_1\wedge C_2 \wedge \ldots C_p,\end{aligned}$$

where each $C_i$ is a disjunction of literals, and an expression

$$\begin{aligned}\phi =Q_1x_1Q_2x_2\ldots Q_nx_nF,\end{aligned}$$

where each $Q_j$ is either $\exists$ or $\forall$, determine whether $\phi$ is true.

### Theorem 7

(Schaefer [23]) QSAT is PSPACE-complete.

Moreover, by trivially adding dummy variables, the following variant of QSAT, which is well-known and which we will call the *Alternating* QSAT problem, is also PSPACE-complete. Note that we assume that the quantifiers alternate and that the number of quantifiers is even.

### Definition 8

(*Alternating QSAT*) Given a set of boolean variables $x_1,\ldots ,x_n$, where *n* is an even non-negative integer, a boolean formula

$$\begin{aligned}F=C_1\wedge C_2 \wedge \ldots C_p,\end{aligned}$$

where each $C_i$ is a disjunction of literals, and an expression

$$\begin{aligned}\phi =Q_1x_1Q_2x_2\ldots Q_nx_nF,\end{aligned}$$

where $Q_j\equiv \exists$ for all odd *j* and $Q_j\equiv \forall$ for all even *j*, where $j\in {\mathbb {N}}$, $1\le j\le n$, determine whether $\phi$ is true.

### Corollary 9

Alternating QSAT is PSPACE-complete.

Before proceeding with the proof of the PSPACE-completeness of the scoring variant of the orthogonal colouring game, we give some intuition on how reductions from Alternating QSAT to games work in general, and how it will apply to our reduction. We suggest that the reader reads this section first, and then refers back to it after going through the construction of $G'$ in the proof.

To be precise, in what follows, a truth value is either True (*T*) or False (*F*). So, what one is looking for is: does there exist a truth value (*T* or *F*) for $x_1$ such that for all truth values (*T* and *F*) for $x_2$, there exists a truth value (*T* or *F*) for $x_3$ such that for all truth values (*T* and *F*) of $x_4$, etc. such that the formula *F* is true.

For $\phi$ to be true, it is equivalent to a two-player game where the player (player 1, let’s say) who wants to make $\phi$ true decides the values of the variables with the “there exists” quantifiers and the adversary (player 2, let’s say) who wants to make $\phi$ false decides the values of the variables with the “for all” quantifiers, and they do it in order; that is, from left to right, and thus, in increasing order of index of the variables.

If $\phi$ is true, then player 1 can always find a truth value for his variable such that whatever player 2 chooses as the truth value for her variable, player 1 can find a truth value for his next variable such that $\ldots$, and so on, such that $\phi$ is true.

If $\phi$ is false, then it means that, at some point, player 2 can choose a truth value for her variable that will make it impossible for $\phi$ to be true and/or player 1 will not be able to find a truth value for his variable to make $\phi$ true.

In our reduction, the Bob gadgets correspond to the variables that player 1 chooses the truth values for, and the Alice gadgets correspond to the variables that player 2 chooses the truth values for. The players are “roughly” forced to colour vertices in their gadgets, which corresponds to choosing the truth value of the associated variable. The gadgets are of decreasing size to ensure that the players play in the same order given by the formula. Also, especially in Bob gadgets, we ensure that Alice has to play again in the same gadget, which forces Bob to play in the same one again, so that now it is Alice’s turn and she wants to play in her gadget. It is not exactly the same for the Alice gadgets because of the difference between ensuring vertices can be coloured and ensuring vertices cannot be coloured.

The idea at the end is that if $\phi$ is true, then the clause vertices cannot be coloured, and so, Bob wins (player 1 in the general setting described above). Otherwise, $\phi$ is false, and so, at least one clause is false, and thus, the clause, which is represented by a set of *k* vertices, can be coloured, and hence, Alice wins (player 2 in the general setting described above).

### Proof of Theorem 2

Since the number of turns and possible plays is bounded above by the number of uncoloured vertices remaining, the problem $MOC_m(G,c_A,c_B)$ is in PSPACE. To show the problem is PSPACE-hard, we reduce from the Alternating QSAT problem where *n* is even. Then the result follows from Corollary 9. The proof is done for the case $m=3$, and it is straightforward to generalise it to any $m>3$. The three colours used are called *T*, *F*, and *X*, with *T* and *F* corresponding to “true” and “false”, respectively.

**Fig. 3:**
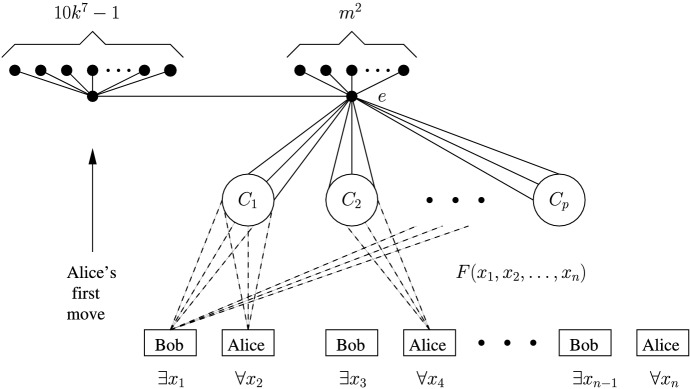
The general construction of *G* from an Alternating QSAT instance using the clause sets $C_i$ and Bob and Alice gadgets. The Bob and the Alice gadgets are connected to the clause sets according to the formula *F*, but note that the details of this construction are more complicated: only a certain subset of the vertices of the Bob or Alice gadget corresponding to variable $x_j$ is connected to the clause sets in which $x_j$ appears, and another subset of vertices is connected to the clause sets in which ${\overline{x}}_j$ appears, with the rest of the vertices in the gadget not being connected to any clause set. See the details of the construction at the beginning of the proof of Theorem 2. This construction is made to guarantee that, in the first phase of the game, the players are forced to play in the Alice and Bob gadgets from left to right (three moves in a Bob gadget by Bob, Alice, and then Bob again, and one move in an Alice gadget by Alice) and not to play in a clause set, unless they would like to lose immediately. See Figs. 4 and 5 for the details of the Bob and Alice gadgets

**General idea of the construction of** *G*. We first describe the construction of *G* from an instance of the Alternating QSAT problem and recall that *n* is even. Let $G_A$ ($G_B$, respectively) be Alice’s (Bob’s, respectively) copy of *G*. Note that in general, for any vertex $v\in V(G)$, since we have partial colourings, we can add pre-coloured vertices of degree 1 adjacent to *v* so as to restrict the colours that can be given to *v*. By forbidding all colours but *T*, *F*, and *X* in this way, the proof can be generalised to any $m>3$, and so, as mentioned before, we focus on the case $m=3$. From here on, when we say that only certain colours can be given to a vertex *v*, it is implied that we have added the vertices of degree 1 adjacent to *v* and given them the appropriate colours in the same copy of *G*. First, start with a star with $m^2+1$ leaves such that its centre is the vertex *e* and colour the first $m^2$ leaves in both copies of *G* such that all permutations of any two colours are used in terms of orthogonality. From here on, for all new vertices that are not pre-coloured in $G_A$, we make them uncolourable in $G_B$ (by forbidding every colour). For the last uncoloured leaf, for some $k\in {\mathbb {N}}$ such that $k>\max (n,m,p)$, add $10k^7-1$ vertices of degree 1 adjacent to it that can only be coloured *X*. For each clause $C_i \in \{C_1,\dots ,C_p\}$, add an independent set of size *k*, that we will call the clause set $C_i$. Make *e* adjacent to all the vertices of the clause sets (this is just to guarantee that the graph we construct is connected) and note that *e* is not colourable. Also, make it so that all the vertices of the clause sets can only be coloured *T*. Let us now define two gadgets for the variable vertices $x_1,\ldots ,x_n$. See Fig. 3 for an illustration of the general idea of the construction of *G*.

**Fig. 4:**
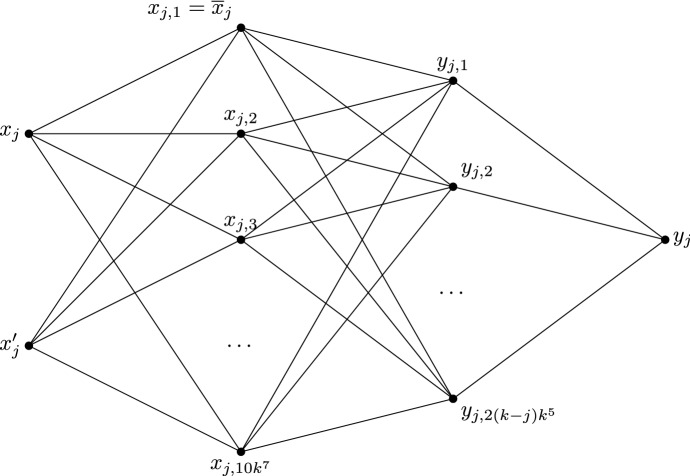
A Bob gadget

**Gadgets—Bob gadget.** First, we define the variable gadget for Bob, called the Bob gadget. For each $j\in \{1,\ldots ,n\}$ such that *j* is odd, there is a Bob gadget constructed as follows. There are four independent sets $S_{j,1}$, $S_{j,2}$, $S_{j,3}$, and $S_{j,4}$ of sizes 2, $10k^7$, $2(k-j)k^5$, and 1, respectively. For each *i* in $\{1,2,3\}$, each vertex of $S_{j,i}$ is adjacent to every vertex of $S_{j,i+1}$. Denote by $x_j$ and $x_j'$ the vertices of $S_{j,1}$, by $x_{j,1}$, ..., $x_{j,10k^7}$ the vertices of $S_{j,2}$, by $y_{j,1}$, ..., $y_{j,2(k-j)k^5}$ the vertices of $S_{j,3}$, and by $y_j$ the vertex of $S_{j,4}$. Let ${{\overline{x}}}_j = x_{j,1}$. For each $1\le i\le p$ such that the literal $x_j$ is in the clause $C_i$, make the vertex $x_j$ adjacent to all the vertices of $C_i$, and, for each $1\le i\le p$ such that the literal ${{\overline{x}}}_j$ is in the clause $C_i$, make the vertices $x_{j,1}$, $x_{j,2}$, ..., $x_{j,k^2}$ adjacent to every vertex of $C_i$. Finally, make it so that $x_j$ is colourable with *X*, *T* or *F*, and all other vertices in the gadget are only colourable with *T* or *F*. See Fig. 4.

**Fig. 5:**
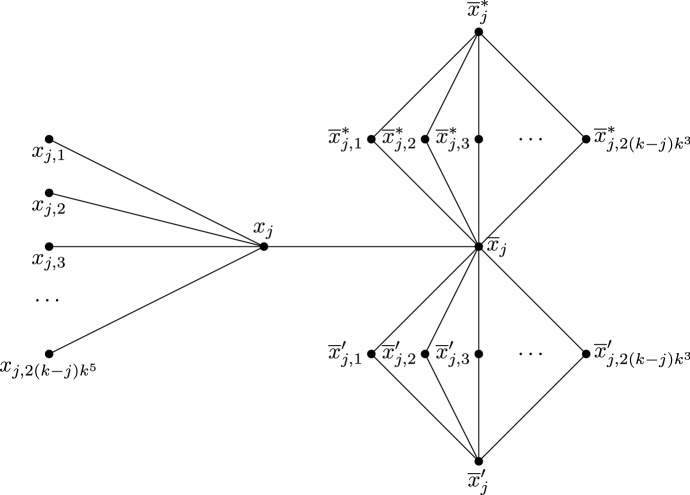
An Alice gadget

**Gadgets—Alice gadget.** Secondly, we define the variable gadget for Alice, called the Alice gadget. For each $j\in \{1,\ldots ,n\}$ such that *j* is even, there is an Alice gadget constructed as follows. There are two vertices $x_j$ and $\overline{x}_j$, such that $x_j$ is adjacent to $2(k-j)k^5$ vertices $x_{j,1},\ldots ,x_{j,2(k-j)k^5}$ and to ${\overline{x}}_j$. Moreover, ${\overline{x}}_j$ is one of the two vertices in the maximal independent set of size 2 of two $K_{2,2(k-j)k^3}$’s that are vertex-disjoint except for ${\overline{x}}_j$, with ${\overline{x}}_j'$ (${\overline{x}}_j^*$, respectively) being the other vertex in the maximal independent set of size 2 of the first (second, respectively) $K_{2,2(k-j)k^3}$. Let ${\overline{x}}'_{j,1},\ldots ,{\overline{x}}'_{j,2(k-j)k^3}$ (${\overline{x}}^*_{j,1},\ldots ,{\overline{x}}^*_{j,2(k-j)k^3}$, respectively) be the $2(k-j)k^3$ vertices in the maximal independent set of size $2(k-j)k^3$ of the first (second, respectively) $K_{2,2(k-j)k^3}$. For each $1\le i\le p$ such that the literal $x_j$ is in the clause $C_i$, make the vertex $x_j$ adjacent to every vertex of $C_i$, and, for each $1\le i\le p$ such that the literal ${{\overline{x}}}_j$ is in the clause $C_i$, make ${{\overline{x}}}_j$ adjacent to every vertex of $C_i$. Finally, make it so that $x_j$ is colourable with *X*, *T* or *F*, the vertices $x_{j,1},\ldots ,x_{j,2(k-j)k^5}$ are only colourable with *X*, the vertices ${\overline{x}}'_{j,1},\ldots ,{\overline{x}}'_{j,2(k-j)k^3}$ (${\overline{x}}^*_{j,1},\ldots ,{\overline{x}}^*_{j,2(k-j)k^3}$, respectively) are only colourable with *T* (*F*, respectively), and all other vertices in the gadget are only colourable with *T* or *F*. See Fig. 5. The graph *G* constructed so far is depicted schematically in Fig. 3.

**Refining the construction.** More pre-coloured leaves adjacent to *e* may be added to either of the copies of *G* to ensure there are exactly

$$\begin{aligned} \alpha = 10k^7+k-1+\frac{n}{2}(10k^7+3)+\sum \limits _{j\text { even, }j\le n}(2(k-j)k^5+2(k-j)k^3+3) \end{aligned}$$

more vertices coloured in $G_B$. Essentially, $\alpha$ corresponds more or less to the number of vertices that are not coloured, but will be at the end of the game. Those vertices are those of the large star with $10k^7$ neighbours (one of which is *e* and is not colourable), the vertices in $S_{j,1}$, $S_{j,2}$, and $S_{j,4}$ for each Bob gadget, which totals to $10k^7+3$ vertices for each Bob gadget, and the vertices $x_j$, $x_{j,i}$, ${\overline{x}}_j'$, ${\overline{x}}_j^*$, and either the ${\overline{x}}_{j,i}'$ or the ${\overline{x}}_{j,i}^*$ for each Alice gadget, which totals to $2(k-j)k^5+2(k-j)k^3+3$ or $2(k-j)k^5+2(k-j)k^3+4$ (since one vertex may not be coloured, and hence, why we say more or less) for the Alice gadget *j* for each even $j\le n$. To this, $k-1$ vertices are added, so that $\alpha$ vertices may always be coloured if a clause set can always be coloured, and so, that $\alpha$ vertices can never be coloured if a clause set cannot be coloured. The construction of *G* is now complete and it is clearly achieved in polynomial time.

**Winning strategy for Bob if and only if** $\phi$ **is true.** We will now show that $\phi$ is true if and only if Bob has a winning strategy in $G_A\cup G_B$. We will also show that there cannot be an equality, and thus, that $\phi$ is false if and only if Alice has a winning strategy in $G_A\cup G_B$.

**Outline of the proof of the equivalence above.** The game will be split into two phases called the *first* and *second* phase, respectively, with all the turns of the first phase coming before all the turns of the second phase, as expected. We will describe strategies for Alice and Bob for both phases, which we will call *standard*. These strategies are optimal in terms of the outcome of the game and not necessarily the scores at the end, i.e., any strategy for a player is considered optimal if it results in that player winning even if there exists another strategy that results in that player winning with an even higher score or a larger difference in score. The first phase consists of the players playing in each of the gadgets until all gadgets have at least one coloured vertex in them, and in doing so, essentially assigning truth values to the variables $x_1,\ldots ,x_n$ with Bob assigning the truth values to the $x_j$ where *j* is odd and Alice to the $x_j$ where *j* is even, $1\le j\le n$. The structure of *G* ensures that, in the first phase, the players have to play on the Alice and Bob gadgets corresponding to the variables $x_j$ in ascending order of the index *j*. See Fig. 3. The second phase consists of the players finishing colouring the rest of the colourable vertices with the strategies being simpler in this phase. Finally, it is shown that both the players’ strategies are optimal, giving the desired result.

**Standard strategies for the first phase.** Let us consider some strategies for Alice and Bob, and we will try to find the best strategies possible; that is, the strategy that colours as many vertices in $G_A$ as possible for Alice and as few as possible for Bob. Recall that no more vertices in $G_B$ may be coloured, so the following all takes place in $G_A$. When referring to the number of coloured vertices from here on in the proof, we refer to the number of vertices coloured not including the precoloured vertices. The strategy will ensure that at most $\alpha -1$ uncoloured vertices of $G_A$ will become coloured. At the start, there are

$$\begin{aligned} \alpha +k(p-1)+1+ \sum \limits _{j\text { odd, }j\le n}(2(k-j)k^5) + \sum \limits _{j\text { even, }j\le n}(2(k-j)k^3+1) \end{aligned}$$

uncoloured vertices that are colourable. Alice cannot allow $10k^7-1$ of them to become uncolourable, otherwise, she loses immediately. Therefore, Alice must colour the center of the star with $10k^7$ neighbours (one of which is *e*) with either *T* or *F* (or one of the neighbours, that is not *e*, with *X*) on her first turn as otherwise, Bob will colour it *X*, rendering $10k^7-1$ vertices uncolourable.

For a colour $C\in \{T,F\}$, we define the *complementary colour* as

$$\begin{aligned}{\overline{C}}:=\left\{ \begin{array}{ll} F&{}\text {if}\ C=T,\\ T&{}\text {if}\ C=F.\\ \end{array}\right. \end{aligned}$$

We call a Bob gadget *free* if either no one coloured in it or if Bob coloured in the gadget once and Alice coloured in it after Bob. An Alice gadget is *free* if no one coloured in it. Let us first describe a strategy for Bob. While there is a free gadget, consider the first such gadget *j*.

- Suppose first that this gadget is a Bob gadget. If the gadget is uncoloured, then Bob colours a vertex of $S_{j,1}$ with $C\in \{T,F\}$. Note that he can always at least colour $x_j$ with *F* since the vertices of the clause sets can only be coloured *T*. If Alice does not then colour a vertex of $S_{j,2}$ with the complementary colour ${\overline{C}}$, then Bob can colour a vertex of $S_{j,3}$ (if Alice did not colour $y_j$) or the remaining vertex of $S_{j,1}$ (if she did colour $y_j$) with ${\overline{C}}$, thus, rendering the $10k^7$ vertices of $S_{j,2}$ uncolourable. In that case, Alice has already lost since there are too few colourable vertices remaining. Thus, we can assume that Alice colours a vertex of $S_{j,2}$ with ${\overline{C}}$, and then, in the case of a free Bob gadget with exactly two coloured vertices, Bob colours $y_j$ with colour *C*.
- Suppose now that the gadget is an Alice gadget. Bob colours $x_j$ with *X*.

Let us now describe a strategy for Alice. We distinguish three cases.

**Case 1:** Whenever Bob colours a vertex in a Bob gadget *j* where Alice did not colour any vertex yet, she answers by colouring a vertex of $S_{j,2}$. If the vertex coloured by Bob is $y_j$ or a vertex of $S_{j,2}$, then she gives the same colour to the vertex of $S_{j,2}$ she colours. Note that this can always be done since most of the vertices of $S_{j,2}$ (all except the first $k^2$) are not adjacent to any vertex outside of the gadget.

**Case 2:** Whenever Bob colours a vertex in a Bob gadget such that the sole move made by Alice in the gadget was to colour $x_j$ with *X*, she does the following: If Bob colours $x_j'$ ($y_j$, respectively) with colour $C\in \{T,F\}$, then Alice answers by colouring $y_j$ ($x_j'$, respectively) with the complementary colour ${\overline{C}}$, in which case every vertex of the gadget except the first $k^2$ vertices of $S_{j,2}$ can no longer become uncolourable. If Bob colours a vertex of $S_{j,2}$ ($S_{j,3}$, respectively) with colour $C\in \{T,F\}$, then Alice answers by colouring a vertex of $S_{j,3}$ ($S_{j,2}$, respectively) with the complementary colour ${\overline{C}}$, and again every vertex of the gadget except the first $k^2$ vertices of $S_{j,2}$ can no longer become uncolourable.

**Case 3:** Whenever Bob does not do such a move as in Case 1 or Case 2, consider the free gadget *j* with the smallest number *j*.

- Suppose first that this gadget is a Bob gadget. If no vertex of gadget *j* has been coloured, then Alice colours $x_j$ with *X*. If exactly two vertices of gadget *j* have been coloured, then one of them must be a vertex of $S_{j,2}$ coloured with $C\in \{T,F\}$, and Alice colours a vertex of $S_{j,3}$ with the complementary colour ${\overline{C}}$. Note that this is possible since in an optimal strategy, Alice has never coloured $y_j$ since then Bob could have coloured $x_j'$ with the same colour and Alice loses.
- Suppose now that the gadget is an Alice gadget. Alice colours $x_j$ with *T* or *F*, or *X* on one of the $x_{j,i}$’s. Note that at least colouring $x_j$ with *F* is always possible.

We will call the strategies above the *standard strategies* of Alice and Bob.

**Counting.** In the following we will often count those coloured vertices that, at the end of the game, have been coloured by Alice and Bob and, thus, are not the precoloured vertices. We will call them *played coloured vertices*.

**Standard strategies of the first phase are forced.** Let us assume that Bob plays according to his standard strategy. In every gadget *j* where Bob plays first, at least $2(k-j)k^5$ vertices cannot be coloured (the vertices of $S_{j,3}$ for a Bob gadget and the $x_{j,i}$’s for an Alice gadget). Thus, the maximum number of vertices that can theoretically become played coloured vertices is

$$\begin{aligned} \alpha + k(p-1) + 1+ \sum \limits _{j\text { even, j }\le n}(2(k-j)k^3+1) < \alpha + 2k^5. \end{aligned}$$

That value is only obtainable if, other than answering the first moves of Bob in Bob gadgets so as not to lose immediately, Alice always plays on the first gadget where no vertex is coloured, which, in that case, will always be an Alice gadget. If Alice does not always play exactly on those gadgets, then Bob will be able to play first in an earlier gadget than in the previous analysis, and there will be at most

$$\begin{aligned} \alpha - 2k^5 + k(p-1)+ 1 + \sum \limits _{j\text { even, j }\le n}(2(k-j)k^3+1) < \alpha \end{aligned}$$

played coloured vertices in the end, so Alice loses. Therefore, while Bob plays the previous strategy, Alice must answer in Bob gadgets after the first time Bob plays in them, and must answer in the following Alice gadget after the second time Bob plays in a Bob gadget.

Let us now assume that Alice plays according to her standard strategy. In every Bob gadget where Alice plays first, or where Bob played only once before she plays twice, she can make sure that all the vertices except at most $k^2$ (the first $k^2$ vertices of $S_{j,2}$) will eventually be coloured. In Bob gadgets where Bob plays first, she still makes sure that all the vertices except at most $2(k-j)k^5+k^2$ (the first $k^2$ vertices of $S_{j,2}$ and the vertices of $S_{j,3}$) will eventually be coloured. In every Alice gadget where Alice plays first, she can make sure that all the vertices except at most $4(k-j)k^3+1$ (the ${\overline{x}}'_{j,i}$’s, the ${\overline{x}}^*_{j,i}$’s, and ${{\overline{x}}}_j$) will eventually be coloured. In every Alice gadget where Bob plays first, at least four vertices will still eventually be coloured (in the worst case, $x_j$, ${{\overline{x}}}_j$, ${{\overline{x}}}_j'$, and ${{\overline{x}}}_j^*$). Therefore, if Bob plays first in every Bob gadget, and plays a second time in every Bob gadget before Alice plays a second time in them, the number of played coloured vertices will be at least

$$\begin{aligned} \alpha - k + 1 -\frac{n}{2}k^2 - \sum \limits _{j\text { even, j }\le n} (2(k-j)k^3) > \alpha - 2k^5. \end{aligned}$$

Again, if Bob does not do that, the number of played coloured vertices will be at least

$$\begin{aligned} \alpha + 2k^5 - k + 1 -\frac{n}{2}k^2 - \sum \limits _{j\text { even, j }\le n} (2(k-j)k^3) > \alpha , \end{aligned}$$

and Alice wins.

We will call the strategies above the *standard strategies* of Alice and Bob. Until no gadget is free, if Bob plays his standard strategy, then Alice always needs to either directly answer Bob in a Bob gadget, or to play in the first free gadget. Similarly, until no gadget is free, if Alice plays her standard strategy, then Bob always needs to play in the first free gadget.

**Optimality of standard strategies in the first phase.** Assume that Alice and Bob played according to their standard strategies until the current move. Note that this implies that no vertex of a clause set has been coloured up until now. We consider two cases.

- It is Bob’s turn, the first free gadget *j* is thus a Bob gadget. Assume no one played in it. If Bob plays *X* on $x_j$, since that is Alice’s standard strategy, the situation would be as if Alice played first in the gadget. She can then continue with her standard strategy and win. If Bob plays in $S_{j,2}$ or $S_{j,3}$, then Alice can play on the other one, making sure that every vertex of the gadget except $k^2$ vertices will eventually be coloured, as if Alice played first in this gadget using her standard strategy. Alice can then proceed with her standard strategy, and again she wins. Therefore, Bob cannot play on $S_{j,2}$ or $S_{j,3}$. If he colours $y_j$, Alice can answer by colouring a vertex in $S_{j,2}$ with the same colour, at which point the situation is similar to what it would have been if Bob first played on $x'_j$ (Bob can colour a vertex of $S_{j,2}$ with the other colour to make sure the vertices of $S_{j,3}$ are uncolourable, and if he does not, then Alice can colour a vertex of $S_{j,3}$ to make sure all of these vertices will eventually be coloured). We can thus assume that Bob colours a vertex in $S_{j,1}$ with *F* or *T*, and that Alice answers in $S_{j,2}$ with colour $C\in \{T,F\}$. Lastly, Bob still needs to make a move in the gadget *j* since it is still free, and he needs to play his standard move, that is, colour $y_j$ with the complementary colour ${\overline{C}}$.
- It is Alice’s turn, the first free gadget *j* is thus an Alice gadget, and no one played on it. Alice should not colour $x_j$ with *X* as it is according to Bob’s standard strategy, and Bob can then proceed with his standard strategy and win. If Alice colours neither one of the $x_{j,i}$’s with *X*, nor $x_j$ with *T* or *F*, then Bob can colour $x_j$ with *X*, thus rendering all the $x_{j,i}$’s uncolourable, as if Bob had played first in gadget *j*. Bob can then play his standard strategy and win. Therefore, Alice needs to colour one of the $x_{j,i}$’s with *X* or colour $x_j$ with *T* or *F*.

Therefore, until no gadget is free, Alice and Bob both need to play their standard strategy. Let us call what happens while there is a free gadget the *first phase*. Since the last gadget is an Alice gadget, at the end of the first phase, it’s Bob’s turn. The only remaining vertices that may be colourable and may become uncolourable are the vertices of the clause sets, the first $k^2$ vertices of $S_{j,2}$ for *j* odd, and ${{\overline{x}}}_j$, the ${{\overline{x}}}_{j,i}'$’s, and the ${{\overline{x}}}_{j,i}^*$’s for *j* even. Let us now extend the standard strategies as follows for the *second phase*.

**Standard strategies for the second phase.** Here is the strategy for Bob: while there exists an Alice gadget *j* where the ${{\overline{x}}}_{j,i}'$’s or the ${{\overline{x}}}_{j,i}^*$’s are uncoloured and may still become uncolourable, consider the first such Alice gadget. Bob colours ${{\overline{x}}}_j$, ${{\overline{x}}}_j'$ or ${{\overline{x}}}_j^*$ with an appropriate colour from $\{T,F\}$ to make sure either the ${{\overline{x}}}_{j,i}'$’s or the $\overline{x}_{j,i}^*$’s are uncolourable.

Here is the strategy for Alice: while there exists an Alice gadget *j* where the ${{\overline{x}}}_{j,i}'$’s or the ${{\overline{x}}}_{j,i}^*$’s are uncoloured and may still become uncolourable, consider the first such Alice gadget. Alice colours ${{\overline{x}}}_j$, ${{\overline{x}}}_j'$, ${{\overline{x}}}_j^*$, one of the ${{\overline{x}}}_{j,i}'$’s or one of the ${{\overline{x}}}_{j,i}^*$’s with an appropriate colour to make sure either the ${{\overline{x}}}_{j,i}'$’s or the ${{\overline{x}}}_{j,i}^*$’s will eventually be coloured.

It is clear that colouring only one vertex cannot cause both the ${{\overline{x}}}_{j,i}'$’s and the ${{\overline{x}}}_{j,i}^*$’s to be uncolourable, since that vertex would need to have colour *F* and *T*. Moreover, whatever one vertex has been coloured, it is always possible to colour ${{\overline{x}}}_j'$ or ${{\overline{x}}}_j^*$ so that either the ${{\overline{x}}}_{j,i}'$’s or the ${{\overline{x}}}_{j,i}^*$’s are uncolourable. Lastly, whatever one vertex is coloured, it is always possible to colour one of the ${{\overline{x}}}_{j,i}'$’s or one of the ${{\overline{x}}}_{j,i}^*$’s to make sure either the $\overline{x}_{j,i}'$’s or the ${{\overline{x}}}_{j,i}^*$’s will eventually be coloured.

**Standard strategy in second phase is forced for Alice.** If both Alice and Bob play according to their standard strategy, then the number of vertices that will eventually be coloured is at least $\alpha - k + 1 -\frac{n}{2}k^2$ and at most $\alpha + k(p-1) + 1+ \frac{n}{2}$ (the $\frac{n}{2}$ here is from the $\overline{x_j}$’s of each of the Alice gadgets). If Bob plays the standard strategy, then Alice needs to play the standard strategy, otherwise, there will be at most

$$\begin{aligned}\alpha + k(p-1) + 1+ \frac{n}{2} - 2k^3 < \alpha \end{aligned}$$

played coloured vertices in the end, and Alice loses. Even if Alice plays according to the standard strategy, Bob may play once not according to the standard strategy (since it has one step in advance), and continue to follow the standard strategy. If he does so, then Alice can also play once not according to the standard strategy, and so on. However, since neither player can play two consecutive moves in an Alice gadget without letting the other one play two consecutive moves in a previous Alice gadget, eventually they will both have played once in each Alice gadget (in addition to the first time Alice had already played in each of them during the first phase).

**Winning situations.** If Bob can make sure that $k^2$ vertices in a $S_{j,2}$ of a Bob gadget are not colourable, then at most $\alpha + k(p-1) + 1+ \frac{n}{2} - k^2 < \alpha$ can be coloured, and Bob wins. But Alice can make sure that this never happens. Indeed, when she played in that Bob gadget during the first phase, she played on a vertex of $S_{j,2}$. As at that time, no vertex of a clause gadget had been played, she could have played that move on one of the first $k^2$ vertices, with the only additional effect of preventing those $k^2$ vertices from becoming uncolourable (and preventing any play that would make them uncolourable). Therefore, we can assume that Alice always played on one of the first $k^2$ vertices of the $S_{j,2}$’s, say $\overline{x}_j$ without loss of generality, and thus, that all of these vertices will eventually be coloured.

Thus, the number of vertices that will eventually become played coloured vertices is at least

$$\begin{aligned} \alpha - k+1 \end{aligned}$$

and at most

$$\begin{aligned} \alpha + k(p-1) + 1+ \frac{n}{2}. \end{aligned}$$

Now, if at least one vertex of a clause set is eventually coloured, then all of the vertices of that clause set will eventually be coloured, and the number of played coloured vertices is at least

$$\begin{aligned} \alpha - k+1 + k = \alpha + 1, \end{aligned}$$

and Alice wins. If no vertex of a clause set is eventually coloured, then the number of played coloured vertices is at most

$$\begin{aligned} \alpha - k + 1+ \frac{n}{2} < \alpha , \end{aligned}$$

and Bob wins. Therefore, Bob wins if and only if no vertex in a clause set is eventually coloured, and otherwise, Alice wins.

**Proof that Bob wins if and only if** $\phi$ **is true.** Assume that $\phi$ is true. We will describe a winning strategy for Bob in $G_A\cup G_B$ with this assumption. Bob plays according to the standard strategy (unless Alice does not play according to her standard strategy, in which case Bob wins as shown before), with the following additional specifications. In the first phase, in each Bob gadget, Bob colours the vertex $x_j$ with the colour corresponding to the truth assignment of the variable $x_j$ that ensures $\phi$ is true. For the values of the other $x_j$’s (for *j* even), that correspond to the $\forall$ variables, take the colour that Alice gave to $x_j$ if she coloured $x_j$, and *F* if she did not. In the second phase, in each Alice gadget that Bob colours in for the first time in the second phase according to his strategy, Bob colours ${\overline{x}}_j$ with *T* if that colour is available (i.e., if Alice did not colour $x_j$ with *T* and with *F* otherwise). Finally, Bob continues to colour arbitrarily until no vertex remains colourable.

If Alice does not play according to her standard strategy, then Bob wins, as seen above. Otherwise, note that, in the end, each vertex $x_j$ or ${{\overline{x}}}_j$ will be coloured with the colour that corresponds to the truth assignment of its literal. That is immediate for every case except when Alice coloured a vertex with *X* in an Alice gadget *j* during the first phase. In that case, we fixed that $x_j = F$, and since Bob coloured ${\overline{x}}_j$ with *T* during the second phase, $x_j$ cannot become uncolourable and will eventually be coloured *F*. Now since every clause contains at least one literal that is true, no vertex of a clause set can be coloured, and Bob wins.

Assume now that $\phi$ is false. We will describe a winning strategy for Alice in $G_A\cup G_B$ with this assumption. Alice plays according to the standard strategy (unless Bob does not play according to his standard strategy during the first phase, in which case Alice wins as shown before), with the following additional specification. Consider that the odd variables, which correspond to the $\exists$ variables, have a truth assignment corresponding to the first colour given by Bob to a vertex of gadget *j* during the first phase. In every Alice gadget *j* during the first phase, Alice colours $x_j$ with the colour corresponding to the truth assignment of variable $x_j$ that assures that $\phi$ is false. In the end, Alice continues to colour arbitrarily until no vertex remains colourable.

First, note that because of Alice’s answers to Bob’s plays in the first phase, if $x_j'$ is coloured *F* (*T*, respectively) in a Bob gadget during the first phase, then $x_j$ cannot be coloured *T* (*F*, respectively). Similarly, for all *j* odd, all of the vertices in $S_{j,2}$ will eventually be coloured the same. Therefore, no literal vertex is coloured *T* while the corresponding literal is false. In the end, there is at least one clause that is false, and thus, no vertex adjacent to a vertex of the corresponding clause set will be coloured *T*. Thus, all of the vertices of this set are coloured and Alice wins. $\square$

## Recognising Graphs Admitting a Strictly Matched Involution

In [2], it was proven that, if a graph *G* admits a strictly matched involution, then Bob has a drawing strategy in the scoring variant of the orthogonal colouring game, and thus, Bob has a winning strategy in the normal play variant of the same game. In this section, we show that recognising whether a graph admits a strictly matched involution is NP-complete. Our reduction is from the 1-IN-3SAT problem, which was proven to be NP-complete in [23], and is the following.

### Definition 10

(*1-IN-3SAT*) Given a collection of clauses $C_1,\dots ,C_m$, with $m >1$, such that each $C_i$ is a disjunction of exactly three literals, determine whether there exist a truth assignment to the variables such that exactly one literal is true in each $C_i$?

### Theorem 11

(Schaefer [23]) 1-IN-3SAT is NP-complete.

Andres et al. [2] proved that a graph *G* admits a strictly matched involution if and only if its vertex set *V*(*G*) can be partitioned into a clique *C* and a set inducing a graph that has a perfect matching *M* such that:

- for any two edges $vw,xy\in M$, the graph induced by *v*, *w*, *x*, *y* is isomorphic to a $2K_2$, a $C_4$ or a $K_4$;
- for any edge $vw\in M$ and any vertex $z\in C$, the graph induced by the vertices *v*, *w*, *z* is isomorphic to a $K_1\cup K_2$ or a $K_3$.

We call such a partition an $({{{\mathcal {M}}}},{{{\mathcal {C}}}})$-partition, where *M* is the set of edges of a (not necessarily induced) matching and *C* is the set of vertices of the clique. Informally, we will say that a vertex is in *M* if it is incident to an edge of *M*. The class of graphs that admit an $({{{\mathcal {M}}}},{{{\mathcal {C}}}})$-partition or, equivalently, the class of graphs that admit a strictly matched involution, is denoted by $\mathcal{M}\mathcal{I}$.

### Theorem 12

The problem of deciding whether a graph is in $\mathcal{M}\mathcal{I}$ is NP-complete.

### Proof

It is clear that the problem is in NP (given the edges of the matching, it is easy to check that we indeed have an $(\mathcal{M},{{{\mathcal {C}}}})$-partition). Let us now prove that it is NP-hard. We reduce from 1-IN-3SAT. Then the result follows from Theorem 11. Recall that we say a vertex is in *M* if it is an end vertex of an edge in *M*.

**Fig. 6:**
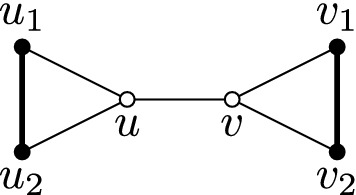
The clique gadget

Let us define three gadgets. These gadgets will impose some specific properties in graphs containing them. First, we define a *clique gadget*, which is the graph depicted in Fig. 6. Note that the clique gadget is in $\mathcal{M}\mathcal{I}$ by putting the white vertices in *C* and the thick edges in *M*. Note further that, for any graph *G* containing the clique gadget such that the black vertices do not have a neighbour outside of the gadget, then in any $({{{\mathcal {M}}}},{{{\mathcal {C}}}})$-partition (*M*, *C*) of *G*, the white vertices are in *C* and the thick edges are in *M*. Indeed, due to non-edges, either $u_1$ and $u_2$ or $v_1$ and $v_2$ (or all four vertices), say $u_1$ and $u_2$, are end vertices of edges in *M*, since their only neighbour besides themselves is *u*, and since $uu_1$ and $uu_2$ cannot both be in *M*, we have $u_1u_2 \in M$, and hence, $u\in C$. Similarly then, $v_1v_2 \in M$ and $v \in C$.

**Fig. 7:**
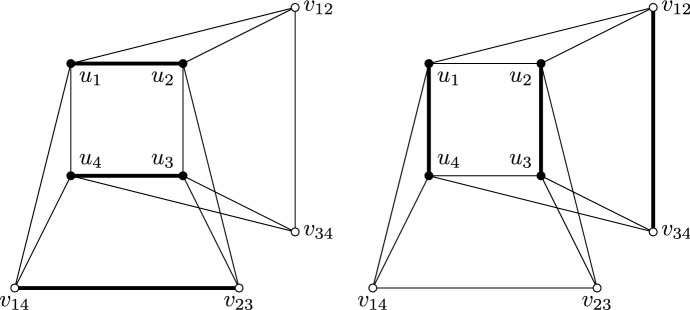
The variable gadget and the two possible sets of edges in *M* (the thick edges)

Let us now define a second gadget, which we will call the *variable gadget*. The gadget is as depicted in Fig. 7. Note that putting the thick edges in any of the two graphs (depicted in Fig. 7) in *M* and the two remaining vertices in *C* leads to an $({{{\mathcal {M}}}},{{{\mathcal {C}}}})$-partition of the gadget. Now assume that a graph *G* contains a copy of the clique gadget and a copy of the variable gadget such that every white vertex of each gadget is adjacent to the white vertices of the other gadget, and the black vertices of each gadget are not adjacent to any vertex of another gadget. Consider an $({{{\mathcal {M}}}},{{{\mathcal {C}}}})$-partition (*M*, *C*) of *G*. As before, the white vertices of the clique gadget are in *C*, so the black vertices of the variable gadget are in *M*. If an edge not incident to two black vertices, say $u_1v_{12}$, is in *M*, since there is exactly one vertex ($u_2$) that is adjacent to $u_1$ and $v_{12}$, then $u_2$ must be in *C*, a contradiction. Therefore, either $u_1u_2$ and $u_3u_4$ are in *M*, or $u_2u_3$ and $u_1u_4$ are in *M*. In the first case, since $v_{14}$ is adjacent to $u_1$ and not to $u_2$, it must be an end vertex of an edge in *M*. Since $v_{23}$ is the only remaining vertex adjacent to both $u_3$ and $v_{14}$ that is not already an end vertex of an edge in *M*, we have $v_{14}v_{23} \in M$, and as $v_{12}$ ($v_{34}$, respectively) is the only vertex adjacent to both $u_1$ and $u_2$ ($u_3$ and $u_4$, respectively), $v_{12}$ and $v_{34}$ are in *C*. This case corresponds to Fig. 7 (left). In the other case, $u_1u_4$, $u_2u_3$, and $v_{12}v_{34}$ are in *M*, and $v_{14}$ and $v_{23}$ are in *C*. That case corresponds to Fig. 7 (right).

**Fig. 8:**
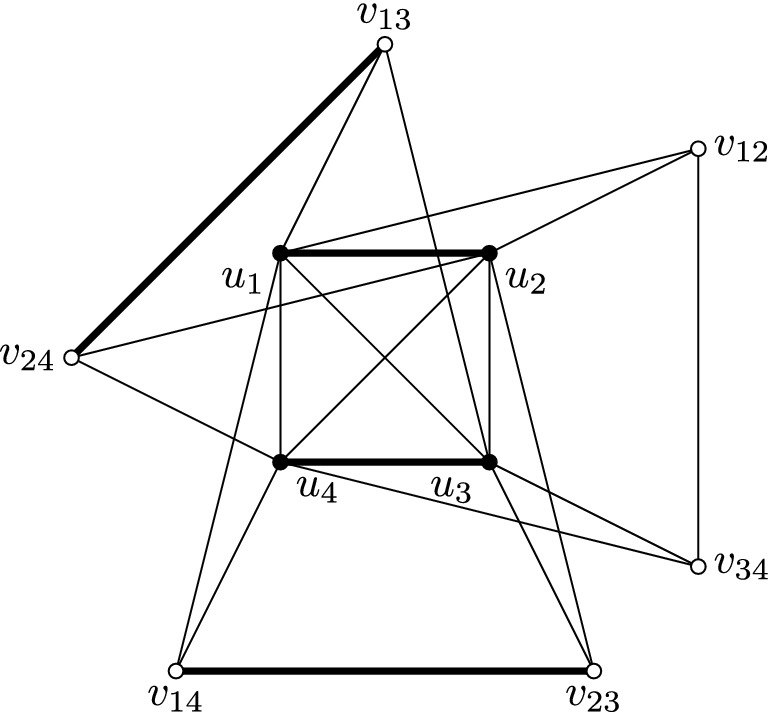
The clause gadget, with one of the three possible sets of edges in *M* (the thick edges)

The third gadget, called the *clause gadget*, is the graph depicted in Fig. 8. It is quite similar to the variable gadget. Defined by the three maximal matchings of the complete graph $K_4$ on the black vertices, we obtain three $(\mathcal{M},{{{\mathcal {C}}}})$-partitions of the clause gadget:

- $u_1u_2,u_3u_4,v_{14}v_{23},v_{13}v_{24}\in M$ and $v_{12},v_{34}\in C$ (see the thick edges in Fig. 8);
- $$u_1u_4,u_2u_3,v_{13}v_{24},v_{12}v_{34}\in M$$ and $$v_{14},v_{23}\in C$$; and
- $$u_1u_3,u_2u_4,v_{12}v_{34},v_{14}v_{23}\in M$$ and $$v_{13},v_{24}\in C$$.

Suppose that there is a graph *G* that contains a clique gadget, some variable gadgets, and a clause gadget, such that the edges between $v_{ij}$’s in the clause gadget are identified to edges between the $v_{ij}$’s in some variable gadgets. Assume further that the white vertices in the clique gadget and the different variable gadgets are all adjacent, and that the black vertices are not adjacent to any vertex outside their respective gadgets. Note that some white vertices in the same clause gadget can be adjacent even if they are not adjacent in Fig. 8. Let us consider an $({{{\mathcal {M}}}},{{{\mathcal {C}}}})$-partition (*M*, *C*) of *G*. As seen previously, each of the edges $v_{14}v_{23}$, $v_{12}v_{34}$, and $v_{13}v_{24}$ is either in *M* or has both of its endpoints in *C*. Since they are not adjacent to the white vertices of the clique gadget, the black vertices of the clause gadget are in *M*, and thus either $u_1u_2$ and $u_3u_4$ are in *M*, or $u_1u_4$ and $u_2u_3$ are in *M*, or $u_1u_3$ and $u_2u_4$ are in *M*. In the first case, we must have $v_{14}v_{23}$ and $v_{13}v_{24}$ in *M*, and $v_{12}$ and $v_{34}$ in *C*, which corresponds to the thick edges in Fig. 8. The two other cases are similar, with respectively $v_{12}v_{34}$ and $v_{13}v_{24}$ in *M* and $v_{14}$ and $v_{23}$ in *C*, and $v_{14}v_{23}$ and $v_{12}v_{34}$ in *M* and $v_{13}$ and $v_{24}$ in *C*.

Consider an instance *I* of 1-IN-3SAT. We will build an equivalent instance of our problem (i.e., the problem of recognising graphs that admit a strictly matched involution). Let *G*(*I*) be the graph obtained as follows:

- Start with a clique gadget.
- Add one variable gadget per variable of *I*, all disjoint and disjoint from the clique gadget, and between any two white vertices of distinct gadgets add an edge. For each variable $x_i$, the literal $x_i$ corresponds to the edge $e(x_i) = v_{14}v_{23}$ and the literal $\bar{x_i}$ corresponds to the edge $e(\bar{x_i}) = v_{12}v_{34}$ in the variable gadget associated to $x_i$.
- For each clause $l_1 \vee l_2 \vee l_3$ of *I* with
  $$\begin{aligned}l_1,l_2,l_3\in \{x_1,\ldots ,x_n,\overline{x_1},\ldots ,\overline{x_n}\},\end{aligned}$$
  add a copy of the clause gadget such that the black vertices are new vertices, the edge $v_{14}v_{23}$ is identified to the edge $e(l_1)$, the edge $v_{12}v_{34}$ is identified to the edge $e(l_2)$, and the edge $v_{13}v_{24}$ is identified to the edge $e(l_3)$.

If *I* is true, then there is a valuation of the variables of *I* that validates *I*. For each true variable in this valuation, pick $v_{12}v_{34}$ in *M* in the corresponding variable gadget, and, for each false variable in this valuation, pick $v_{14}v_{23}$ in *M* in the corresponding variable gadget. This imposes the other edges that are in *M* in each variable gadget. Indeed, as was explained earlier, and as can be seen in Fig. 7, if $v_{12}v_{34}$ is in *M*, then $u_2u_3$ and $u_1u_4$ must also be in *M* and $v_{14}v_{23}$ must be in *C* for there to exist an $(\mathcal{M},{{{\mathcal {C}}}})$-partition of *G*(*I*). The edges in *M* in the clique gadget are trivially imposed as can be seen in Fig. 6. Now in each clause gadget, there are exactly two literals that are false, and their corresponding edges are in *M*, while the edge corresponding to the last literal is not in *M*. Thus, *M* can also be extended to each clause gadget. Now, in the resulting matching *M*, the adjacencies inside the gadgets have already been checked. Note that each edge of *M* has its two endpoints with the same colour (black or white) and in the same gadgets. Therefore, both endpoints of each edge of *M* have the same adjacencies to other gadgets. For the vertices that are not in *M*, they are all white vertices so they are all adjacent, since we never have the vertices of a literal and of its negation. Thus, we truly have an $(\mathcal{M},{{{\mathcal {C}}}})$-partition of *G*(*I*).

Suppose now that there exists an $({{{\mathcal {M}}}},{{{\mathcal {C}}}})$-partition (*M*, *C*) of *G*(*I*). As shown previously, there is exactly one edge in *M* among the $v_{ij}$’s of each variable gadget. If that edge is $v_{12}v_{34}$, then we pick the corresponding variable to be true, and otherwise, we pick it to be false. Now the clause gadgets imply that there is exactly one true literal in each clause. This completes the proof of the theorem. $\square$

## Conclusion and Further Work

We have proven that the normal play (scoring, respectively) variant of the orthogonal colouring game introduced by Andres et al. [2] is PSPACE-complete when partial colourings are part of the input for $m\ge 1$ ($m\ge 3$, respectively) colours. Moreover, the proof of the PSPACE-completeness of the scoring variant can be easily altered to prove that the same colouring scoring game played just on one graph *G*, and thus, without the orthogonality condition, is also PSPACE-complete, which may be of interest to the reader. In the *misère variant* of a combinatorial game, the first player who cannot colour a vertex wins. We have not studied the misère variant of the orthogonal colouring game, but this could be an interesting direction for future work. We have also shown that recognising graphs that admit a strictly matched involution is NP-complete. Lastly, the complexity of the very much related colouring construction game introduced by Bodlaender [9] is still unknown when *k* is a fixed constant, and has been for almost 30 years now. With this in mind we give the following open problems.

### Problem 1

Determine the complexity of the scoring variant of the orthogonal colouring game when partial colourings are given for $m=1$ ($m=2$, respectively) colours.

### Problem 2

Determine the complexity of both variants of the orthogonal colouring game when no partial colouring is given initially.

### Problem 3

For which class of graphs does Alice or Bob have a winning strategy in the misère variant of the orthogonal colouring game and what is the complexity of this variant?

### Problem 4

(Bodlaender [9]) Determine the complexity of the colouring construction game when *k* is a fixed constant.
